# Boerhaave Syndrome With Concomitant Duodenal Perforation: A Rare Case Complicated by Mediastinitis and Empyema

**DOI:** 10.7759/cureus.110207

**Published:** 2026-06-03

**Authors:** Fotini Ampatzidou, Maria Papaioannou, Athanasios Xakis, Anastasia Argyropoulou, Athina Lavrentieva

**Affiliations:** 1 1st Intensive Care Unit, General Hospital of Thessaloniki "G. Papanikolaou", Thessaloniki, GRC; 2 2nd Surgery Department, General Hospital of Thessaloniki "G. Papanikolaou", Thessaloniki, GRC

**Keywords:** boerhaave syndrome, critical care, duodenal perforation, empyema, esophageal rupture, septic shock, thoracotomy

## Abstract

Boerhaave syndrome (spontaneous esophageal rupture) remains a surgical emergency with high mortality, particularly when complicated by mediastinitis, pleural empyema, and septic shock. Concomitant intra-abdominal perforation significantly increases morbidity. We report a case of a 61-year-old male patient presenting with hemodynamic instability due to distal esophageal rupture and duodenal perforation. Imaging revealed bilateral pneumothorax, pneumomediastinum, pneumoperitoneum, and extensive pleural effusions. The patient underwent emergent exploratory laparotomy with esophageal repair over T-tube placement, mediastinal lavage, primary duodenal repair, bilateral chest tube placement, decompressive gastrostomy, and feeding jejunostomy. The postoperative course was complicated by septic shock, bilateral pleural empyema requiring re-interventions including thoracotomy and pleurodesis, multidrug-resistant pulmonary infections, axillary vein thrombosis, *Clostridioides difficile *colitis, and prolonged mechanical ventilation. After comprehensive multidisciplinary management in the ICU, gradual respiratory and hemodynamic stabilization was achieved.

## Introduction

Esophageal perforation is a severe and potentially life-threatening condition requiring prompt diagnosis and multidisciplinary management. The main causes include iatrogenic injury, trauma, foreign body ingestion, underlying esophageal disease, and spontaneous rupture. Boerhaave syndrome accounts for 8-56% of cases and results from a sudden rise in intraesophageal pressure, usually after forceful vomiting [1]. Early diagnosis remains challenging but is crucial for survival, as mortality rates decrease significantly when treatment is initiated within the first 24 hours [2].

Perforations are most commonly located in the distal posterolateral esophagus, typically 2-3 cm above the gastroesophageal junction [3]. Leakage of gastric and esophageal contents into the mediastinum and pleural cavity rapidly leads to mediastinal and pleural inflammation, with potentially life-threatening complications such as mediastinitis, empyema, sepsis, and multiorgan dysfunction [4,5].

The coexistence of Boerhaave syndrome with duodenal ulcer perforation is exceedingly rare and may further aggravate systemic inflammation and clinical deterioration [6]. We report a complex case of combined thoracoabdominal perforation requiring multiple surgical interventions and prolonged intensive care management, ultimately resulting in survival.

## Case presentation

A 61-year-old man with a history of chronic alcohol use, gastroesophageal reflux disease, and previous upper gastrointestinal bleeding was transferred to our emergency department from a regional health center three days after symptom onset, which followed repeated episodes of forceful vomiting.

At the time of presentation, the patient was in critical condition. He had ongoing hemodynamic instability requiring dual vasopressor support (norepinephrine 0.3 μg/kg/min and vasopressin 0.04 units/min), respiratory failure, and clinical signs indicative of a systemic inflammatory response. The Acute Physiology and Chronic Health Evaluation II (APACHE II) score was 10, and the Sequential Organ Failure Assessment (SOFA) score was 7 [7,8]. Initial laboratory evaluation showed elevated inflammatory markers, acute respiratory failure, and early renal dysfunction (Table 1).

**Table 1:** Initial laboratory findings on ICU admission.

Parameter	Value	Reference range
White blood cells (×10⁹/L)	3.2	4.0-10.0
Hemoglobin (g/dL)	10.3	12-18
Platelets (×10⁹/L)	282	150-400
C-reactive protein (mg/L)	4.5	<5
Procalcitonin (ng/mL)	17	<0.05
Serum lactate (mmol/L)	1,5	0.5-2.0
Creatinine (mg/dL)	1.34	0.7-1.3
Urea (mg/dL)	75	15-45
Total bilirubin (mg/dL)	0.7	0.2-1.2
Aspartate aminotransferase (U/L)	47	<40
Alanine aminotransferase (U/L)	18	<41
Arterial pH	7.39	7.35-7.45
Partial pressure of oxygen (PaO₂) (mmHg)	110	80-100
Partial pressure of carbon dioxide (PaCO₂) (mmHg)	39	35-45
Bicarbonate (HCO₃⁻) (mmol/L)	22	22-26
Base excess (mEq/L)	-2	-2 to +2

**Figure 1:**
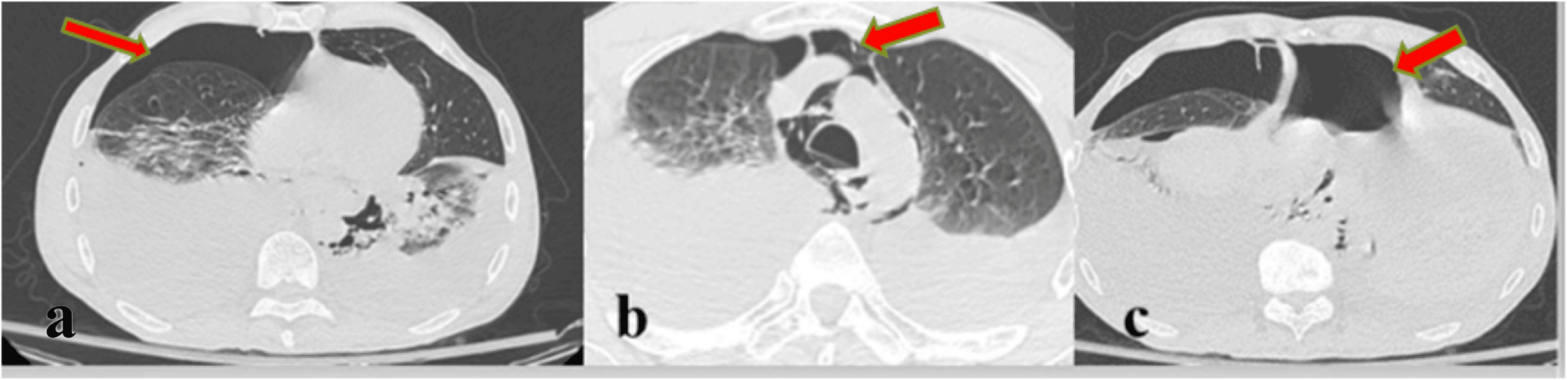
(a) Right pneumothorax (red arrow) and extensive bilateral pleural effusions with compression atelectasis. (b) Pneumomediastinum extending cranially toward the cervical region (red arrow). (c) Free intraperitoneal air with gastric distension and an air-fluid level within the stomach (red arrow).

Contrast-enhanced computed tomography (CT) of the chest and abdomen demonstrated rupture of the distal esophagus, right-sided pneumothorax, extensive bilateral pleural effusions, pneumomediastinum, and pneumoperitoneum (Figure 1).

**Figure 2:**
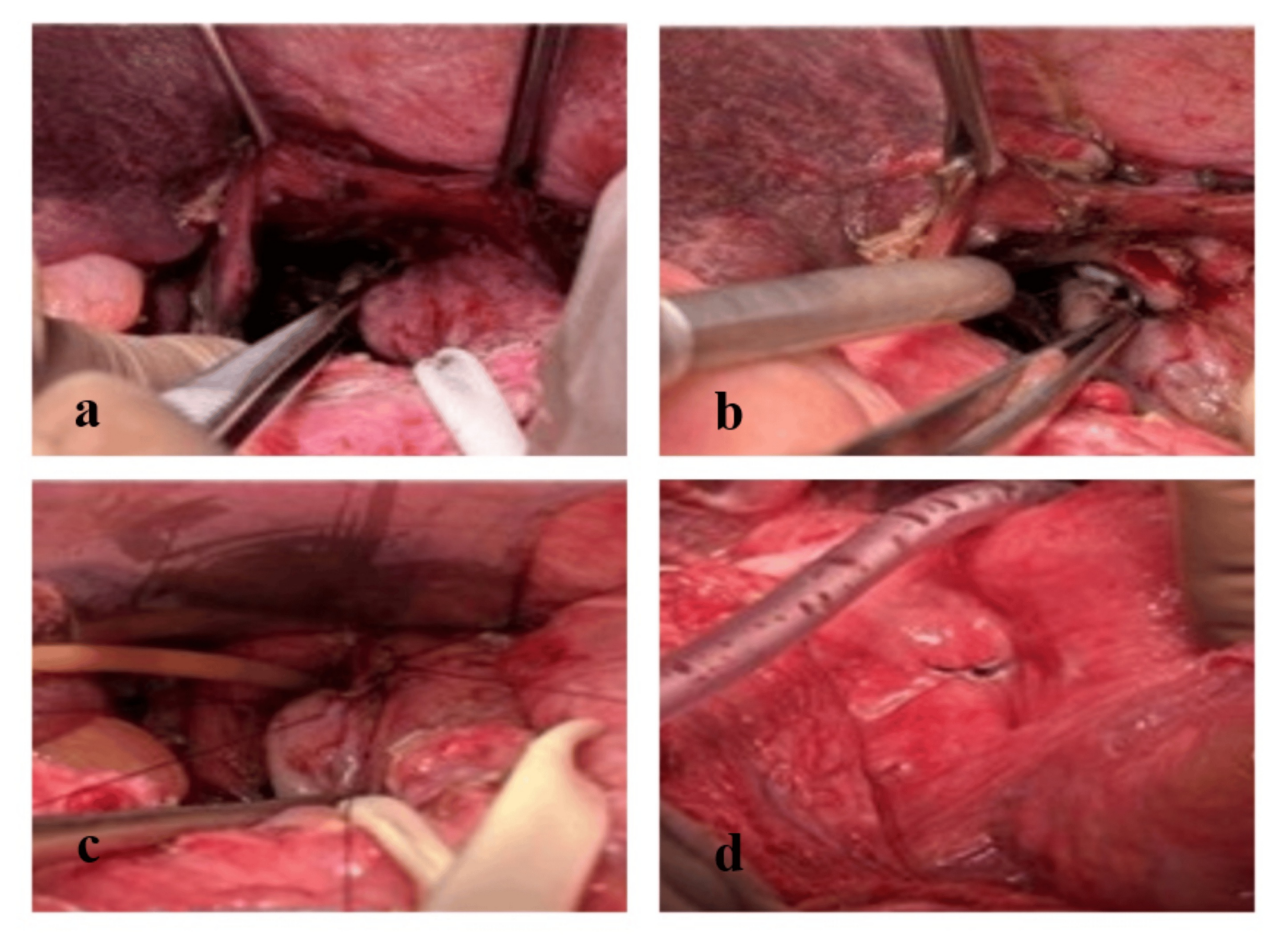
(a) Extensive lower thoracic esophagus mobilization with caudal traction using a Penrose drain. (b) Perforation site in the right lower thoracic esophagus. (c) Esophageal repair over an intraluminal T-tube. (d) Primary repair of the duodenal perforation.

The patient was urgently transferred to the operating room under ongoing hemodynamic support and underwent emergent exploratory laparotomy. Distal esophageal rupture, mediastinal contamination, and concurrent duodenal perforation were verified by intraoperative visual inspection. Surgical management included primary esophageal repair over an intraluminal T-tube, mediastinal drainage, and primary duodenal repair (Figure 2).

**Figure 3:**
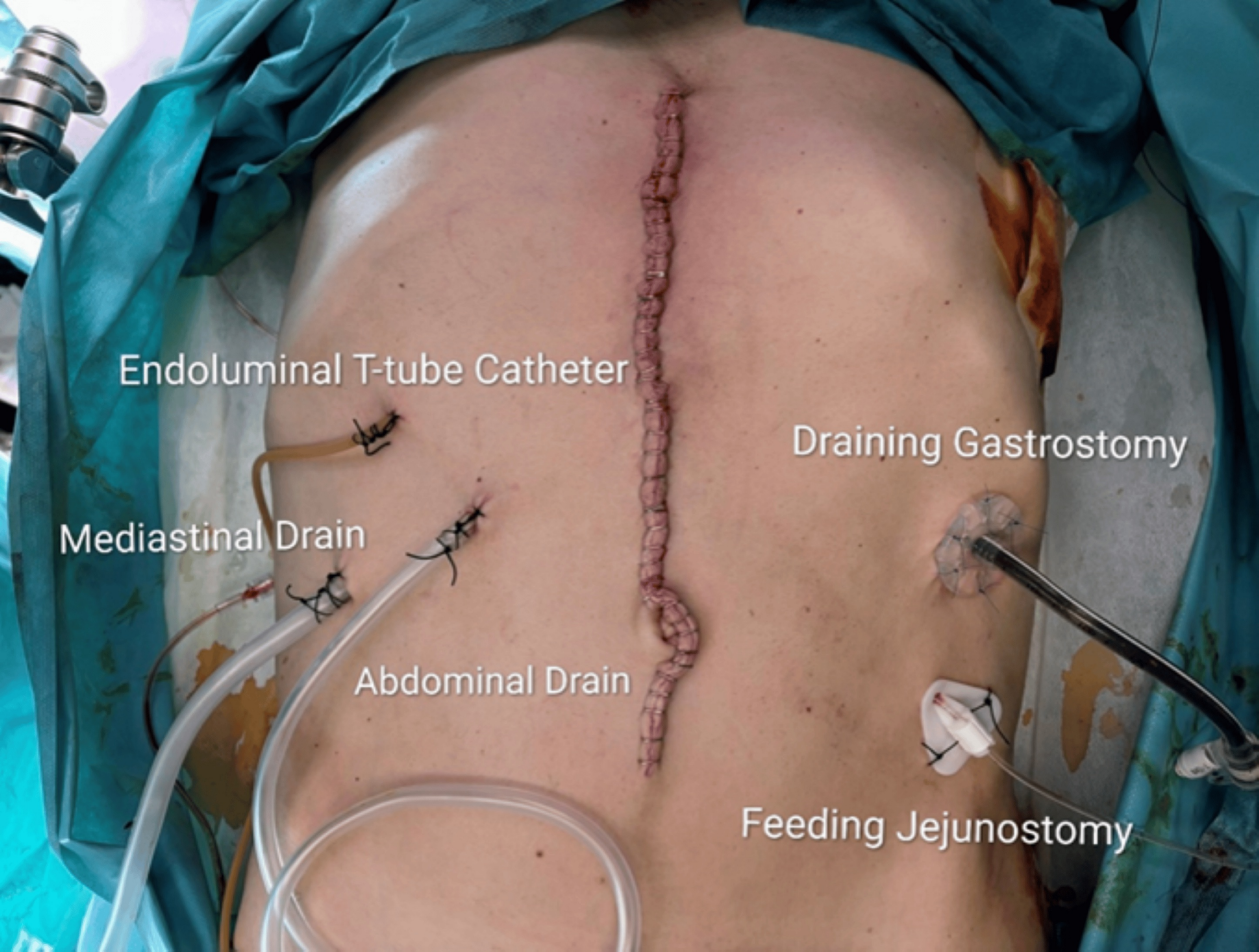
Bilateral chest tubes, decompressive gastrostomy, and feeding jejunostomy.

In addition to esophageal and duodenal repair, bilateral chest tube placement, decompressive gastrostomy, and feeding jejunostomy were performed (Figure 3).

On ICU day 7, a left-sided pleural empyema was identified, requiring a left thoracotomy with pleural and pulmonary decortication, as well as significant debridement. Microbiological samples were collected, and chest tubes were inserted for drainage. 

Cultures of bronchial secretions detected multidrug-resistant organisms, such as *Acinetobacter* spp. and *Stenotrophomonas maltophilia*, whereas pleural fluid from the left-sided empyema isolated *Candida albicans*. Broad-spectrum antibiotic treatment was started empirically and then modified based on microbiological evidence and antimicrobial susceptibility testing.

A right-sided pleural fluid investigation on ICU day 35 revealed an exudative empyema. During the surgical drainage, a purulent accumulation with pseudomembranes was identified. Cultures were collected, warm normal saline was injected into the pleural cavity, and debridement of the parietal pleura was performed. A 32-Fr chest tube was placed.

Moreover, the patient developed *Clostridioides difficile* colitis during hospitalization. During the ICU stay, left axillary vein thrombosis was diagnosed and treated with therapeutic low-molecular-weight heparin, with favorable clinical evolution.

Hemodynamic and respiratory status gradually improved, allowing vasopressor discontinuation and successful weaning from mechanical ventilation. Arterial blood gas analysis demonstrated adequate oxygenation and stable respiratory parameters. The patient was successfully transitioned to spontaneous breathing with supplemental oxygen support.

The serial radiological assessments showed a steady improvement, with the pneumomediastinum and pneumoperitoneum gradually resolving, a noticeable decrease in pleural collections, and better pulmonary parenchymal findings.

At transfer to the surgical ward, the patient was fully awake and hemodynamically stable without vasopressor support. Renal function had normalized, antimicrobial therapy had been discontinued, and enteral feeding through percutaneous endoscopic gastrostomy (PEG) was well tolerated.

## Discussion

Spontaneous esophageal rupture, or Boerhaave syndrome, constitutes a life-threatening surgical condition. Prognosis is determined by the timeliness of diagnosis, the extent of mediastinal and pleural contamination, and the promptness of therapeutic intervention [9]. This case reflects the impact of delayed presentation, dual thoracic and abdominal pathology, and subsequent septic complications, necessitating coordinated, long-term multidisciplinary management.

One of the most important factors of prognosis in esophageal rupture is the time between diagnosis and intervention. Management during the first 24 hours is associated with considerably decreased mortality, but delays after 48 hours are connected to significantly poorer results due to persistent mediastinal and pleural contamination [6]. In our case, the patient presented three days after the symptoms began, which most likely led to the severe mediastinitis, bilateral pleural involvement, and ultimately septic shock.

Distal esophageal rupture and duodenal perforation occurred simultaneously in this case, which is a significant aspect because it is rarely documented [10]. Mediastinitis can be caused by either chemicals or infection when an esophageal rupture occurs alone, but systemic inflammation and septic burden are much more severe when an intra-abdominal perforation is present. The involvement of both the thoracic and abdominal compartments at the same time causes a dual-source septic process, which complicates therapy and increases the risk of multiorgan failure [11].

In Boerhaave syndrome, contamination of the mediastinum quickly leads to infection and septic shock [1]. Bilateral empyema, in our case, highlights the severe progression associated with delayed diagnosis.

There remains considerable debate regarding the optimal management of esophageal perforation. The most appropriate approach depends on the patient’s clinical condition, the site of perforation, and the extent of contamination. In this particular case, emergent laparotomy with primary repair, as opposed to T-tube insertion, mediastinal lavage, and extensive drainage, seemed appropriate due to the patient's hemodynamic instability and concomitant abdominal pathology [12]. Multidisciplinary treatment is important in circumstances like this, which are quite complicated. A favorable outcome relies on collaborative effort between surgeons, intensivists, infectious disease specialists, and microbiologists [13]. The necessity for recurrent operations, such as thoracotomy and decortication, highlights the importance of continuous analysis and aggressive source control.

During the ICU stay, the patient developed multiple complications, including multidrug-resistant infections, *Clostridioides difficile* colitis, and venous thrombosis. These reflect the complex interplay between severe infection, immune dysregulation, and prolonged supportive care. The isolation of pathogens such as *Acinetobacter *spp. and *Stenotrophomonas maltophilia *highlights the need for microbiology-guided antimicrobial therapy, particularly in patients with extended ICU stays [14]. Over time, hemodynamic stability was restored, and the patient was weaned from ventilation, highlighting the potential for recovery even in severe illness.

The given case illustrates several key concepts from a surgical perspective. In addition to proper drainage of the mediastinal and pleural areas, prompt and efficient source control is crucial. If there is a worsening of the clinical condition or if the infection is still present, re-intervention should be considered immediately. Also, a jejunostomy or other enteral nutritional support system is crucial to the healing process [15].

Despite multiple adverse prognostic factors, including delayed diagnosis, septic shock, bilateral empyema, and multidrug-resistant infections, a favorable outcome was achieved with timely surgical intervention, appropriate antimicrobial therapy, and comprehensive intensive care. Existing evidence is limited due to the low occurrence of concurrent esophageal and duodenal perforation; this case adds to the literature and calls for an individualized, aggressive, multidisciplinary approach.

## Conclusions

The coexistence of Boerhaave syndrome with concomitant intra-abdominal perforation is exceedingly uncommon and sparsely reported in the literature. The management of patients with such a complex condition requires a multidisciplinary approach involving collaboration among multiple specialties.
